# *TRIM22* genotype is not associated with markers of disease progression in children with HIV-1 infection

**DOI:** 10.1097/QAD.0000000000003053

**Published:** 2021-12-01

**Authors:** Michael T Boswell, Louis-Marie Yindom, Dan Hameiri-Bowen, Grace Mchugh, Ethel Dauya, Tsitsi Bandason, Hilda Mujuru, Joakim Esbjörnsson, Rashida A Ferrand, Sarah Rowland-Jones

**Affiliations:** 1 Nuffield Department of Medicine, Oxford University, United Kingdom.; 2 Biomedical Research and Training Institute, Zimbabwe; 3 Department of Paediatrics, University of Zimbabwe, Zimbabwe; 4 Department of Clinical Research, London School of Hygiene and Tropical Medicine, United Kingdom; 5 Department of Translational Medicine, Lund University, Sweden

**Keywords:** HIV-1, TRIM22, perinatal infection, stunting, children, disease progression

## Abstract

**Objective:**

Untreated perinatal HIV-1 infection is often associated with rapid disease progression in children with HIV (CWH), characterised by a high viral loads and early mortality. TRIM22 is a host restriction factor which directly inhibits HIV-1 transcription, and with genotype variation reported as associated disease progression in adults. We tested the hypothesis that *TRIM22* genotype is associated with disease progression in CWH.

**Design:**

ART-naïve CWH, aged 6 to 16 years, were recruited from primary care clinics in Harare, Zimbabwe. We performed a candidate gene association study of *TRIM22* genotype and haplotypes with markers of disease progression and indicators of advanced disease.

**Methods:**

*TRIM22* exons three and four were sequenced by Sanger sequencing and single nucleotide polymorphisms were associated with markers of disease progression (CD4+ T cell count and viral load) and clinical indicators of advanced HIV disease (presence of stunting and chronic diarrhoea). Associations were tested using multivariate linear and logistic regression models.

**Results:**

A total of 241 children, median age 11.4 years, 49% female, were included. Stunting was present in 16% of participants. Five SNPs were analysed including rs7935564, rs2291842, rs78484876, rs1063303 and rs61735273. The median CD4+ count was 342 (IQR: 195 - 533) cells/μL and median HIV-1 viral load 34 199 (IQR: 8211 – 90 662) IU/mL. *TRIM22* genotype and haplotypes were not associated with CD4+ T cell count, HIV-1 viral load, stunting or chronic diarrhoea.

**Conclusion:**

*TRIM22* genotype was not associated with markers of HIV disease progression markers or advanced disease in CWH.

## Introduction

Children with HIV-1 (CWH) progress to AIDS early after HIV-1 infection and have high mortality if untreated [1]. In the absence of antiretroviral therapy (ART), approximately 50% of perinatally infected CWH progress to AIDS at one year of age and die by two years [2]. In untreated CWH who survive early childhood there is substantial variation in disease progression, with CD4+ T cell counts and HIV-1 plasma viral load (pVL) serving as independent predictive markers of later progression [3]. In adults, human leucocyte antigen (HLA) and killer immunoglobulin like receptor (KIR) genes have been consistently associated with HIV disease progression rates [4]. However, HLA types do not associate with significant differences in viral replicative capacity (RC), CD4+ T cell counts or pVL in children – nor do they affect disease progression rates [5]. In HIV-1 infected infants, positive selection pressure on cytotoxic T lymphocyte epitopes in *gag* and *nef* is associated with slower disease progression [6]. This weakened effect of HLA types on disease progression in CWH is thought to be caused by pre-adaptation to the parent’s HLA type by the transmitted virus and lower levels of immune activation in the context of higher viral loads [7,8].

Therefore, HLA-independent pathways of viral restriction may be important in CWH, particularly those involved in innate immunity [9]. Significant associations have been found between disease progression rates in CWH and single nucleotide polymorphisms in vitamin D and chemokine receptor genes [10,11]. Deletions in the chemokine receptor gene, *CCR5*, are also associated with slower disease progression [4].

The host restriction factor TRIM22 blocks transcription factor Sp1 from binding to the HIV-1 LTR region, thereby decreasing viral transcription. TRIM22 also prevents viral particle release through its association with Gag proteins [12,13]. Increased *TRIM22* RNA expression in peripheral blood mononuclear cells (PBMC) is associated with higher CD4+ T cell counts and lower HIV-1 pVL in adults [14]. The *TRIM22* gene is highly polymorphic and several single nucleotide polymorphisms (SNPs) alter its protein structure or function [15]. The SNPs which have been associated with clinical phenotypes in relation to HIV-1 and Hepatitis C infection are found in exons three and four [16,17]. These exons are translated into the coiled-coil of TRIM22, the region which is responsible for higher order multimer formation, polyubiquitination and subsequent activation of nucleotide binding oligomerization domain containing 2 (NOD-2) [18,19]. Combinations of non-synonymous SNPs which are inherited together (haplotypes) in *TRIM22* have also been linked to HIV-1 disease progression in adults [16]. In an Italian cohort a haplotype of two SNPs in exon four (rs7935564(A) and rs1063303(C)) was associated with long-term non-progression in HIV-1 infected adults [16].

A pathological loss of function mutation in this region of TRIM22, rs61735273(T) is associated with dysregulation of NOD-2-dependent activation of interferon-beta signalling and NF-κB. Disruption of this pathway can result in severe inflammatory bowel disease in children [18].

To test the hypothesis that *TRIM22* genotype is associated with disease progression in CWH, we performed a candidate gene association study with CD4+ T cell count data, HIV-1 pVL and clinical indicators of advanced HIV disease.

## Methods

### Study participants

Participants were recruited from the **Z**imbabwe study for **En**hancing Testing and **I**mproving **T**reatment of **H**IV in Children (ZENITH) study, which investigated provision of provider-initiated HIV testing and counselling for 6-16 year olds in seven primary care clinics in Harare [20]. All newly diagnosed individuals were recruited into a cohort study. Demographic and clinical data was collected using a questionnaire, and all participants underwent a standardised clinical examination.

Blood samples were collected for CD4+ T cell counts, viral load testing and DNA extraction. Antiretroviral therapy was initiated according to contemporary national guidelines. Here, we analysed data from participants prior to ART initiation. Demographic variables which were analysed included age and sex. Stunting (defined as height-for-age z-score <-2) [17] and chronic diarrhoea (defined as diarrhoea lasting for longer than two weeks) were analysed as indicators of advanced disease [22,23].

CD4+ T cell counts were measured using an Alere PIMA CD4 (Waltham, Massachusetts, USA) machine. Plasma levels of HIV-1 viral RNA were quantified using the RealStar HIV RT-PCR kit version 1.0 (Altona diagnostics, Hamburg, Germany) according to the manufacturer’s instructions.

### DNA extraction and sequencing

Briefly - DNA was extracted from peripheral blood mononuclear cells (PBMCs) using an in-house salting-out protocol. *TRIM22* exons three and four were amplified by polymerase chain reaction (PCR) and sequenced by Sanger sequencing at the Medical Research Council Weatherall Institute for Molecular Medicine (Oxford, United Kingdom). Further details are contained in the supplementary materials (table S1).

### Statistical analysis

Statistical analysis was done in R, version 3.4.0 [24,25]. Figures were prepared using the ggpubr package [26]. Logistic regression models were used to test associations of *TRIM22* genotype with indicators of advanced disease, namely i) CD4+ T cell count less than 200 cells/μL before starting ART, ii) stunting, and iii) chronic diarrhoea. Linear regression models were analysed to determine if *TRIM22* genotype contributed to variation in CD4+ T cell counts when adjusted for age.

### Ethical considerations

Written informed guardian consent and participant assent was obtained prior to enrolment. The Medical Research Council of Zimbabwe, the Biomedical Research Training Institute Institutional Review Board, and the Ethics Committees of Harare City Health Department, Harare Central Hospital, and the London School of Hygiene and Tropical Medicine approved the study.

## Results

A total of 241 participants had DNA samples available and were included in this analysis. This sample was representative of the larger ZENITH cohort in distributions of age, sex, CD4+ T cell counts and HIV-1 pVL (table S2). All participants had been perinatally infected with HIV-1 and all were ART-naïve at the time of sample collection. Median age was 11.4 (IQR: 9.1 – 13.5) years at the time samples were collected (table 1). A total of 15% participants had a HIV-1 pVL less than 1000 IU/mL and 27% of participants had a CD4+ T cell count below 200 cells/μL.

We identified six SNPs in *TRIM22*, all previously reported on the National Center for Biotechnology Information Single Nucleotide Polymorphism database (dbSNP) (table 1). We included *TRIM22* SNPs rs7935564, rs78484876, rs2291842, rs1063303 and rs61735273 in further association studies, as their minor allele frequencies were above 5%.

CD4+ T cell counts and HIV-1 pVL were equivalent between males and females (Mann-Whitney [MW] *P* = 0.82 and 0.99 respectively). Neither stunting nor chronic diarrhoea were associated with differences in CD4+ T cell counts or HIV pVL (MW *P* > 0.05). HIV-1 pVL did not correlate with CD4+ T cell counts (Spearman correlation: ρ = -0.02, *P* = 0.74). There was a weak negative correlation between CD4+ T cell count and age (Spearman correlation: ρ = -0.29, *P* = 6.66x10^-6^). The correlation between HIV-1 pVL and age was not significant (spearman correlation: ρ = -0.11, *P* = 0.12).

There were no significant associations between *TRIM22* genotype and age, CD4+ T cell count or HIV pVL (MW *P* > 0.5 for all pairwise comparisons).

## Age-adjusted CD4+ T cell counts do not vary by *TRIM22* genotype

Age was negatively associated with CD4+ T cell counts (CD4+ T cell count was 37 cells/μL lower with each year of age; 95% CI = 23 to 50 cells/μL; *P* =1.99x10^-7^). *TRIM22* rs1063303 GG genotype was associated with lower CD4+ T cell counts in a multivariate model which adjusted for age (GG vs CC genotype associated with a 92 cells/μL decrease; 95% CI = 3 – 182 cells/μL; *P* = 0.04). However, after removal of three outlier participants with large residuals this association were no longer significant (GG vs CC genotype = -55 cells/μL; -136 to +25 cells/μL; *P* = 0.18).

## *TRIM22* associations with indicators of advanced HIV disease

Older CWH were more likely to have a CD4+ T cell count of less than 200 cells/μL (OR = 1.16 per year, 95% CI = 1.00 – 1.25, *P* = 0.05), but not to be stunted or have chronic diarrhoea (*P* > 0.05). HIV pVL was not associated with indicators of advanced disease (*P* > 0.05). rs61735273 TT genotype was associated with increased odds of chronic diarrhoea (OR 4.33, 95% CI = 0.88 - 16.83, *P* = 0.04) – albeit with weak statistical support. *TRIM22* genotype was not associated with CD4+ T cell counts below 200 cells/ μL or stunting (table S3).

## *TRIM22* haplotypes are not associated with differences in disease progression markers in children living with HIV-1

**Figure 1:**
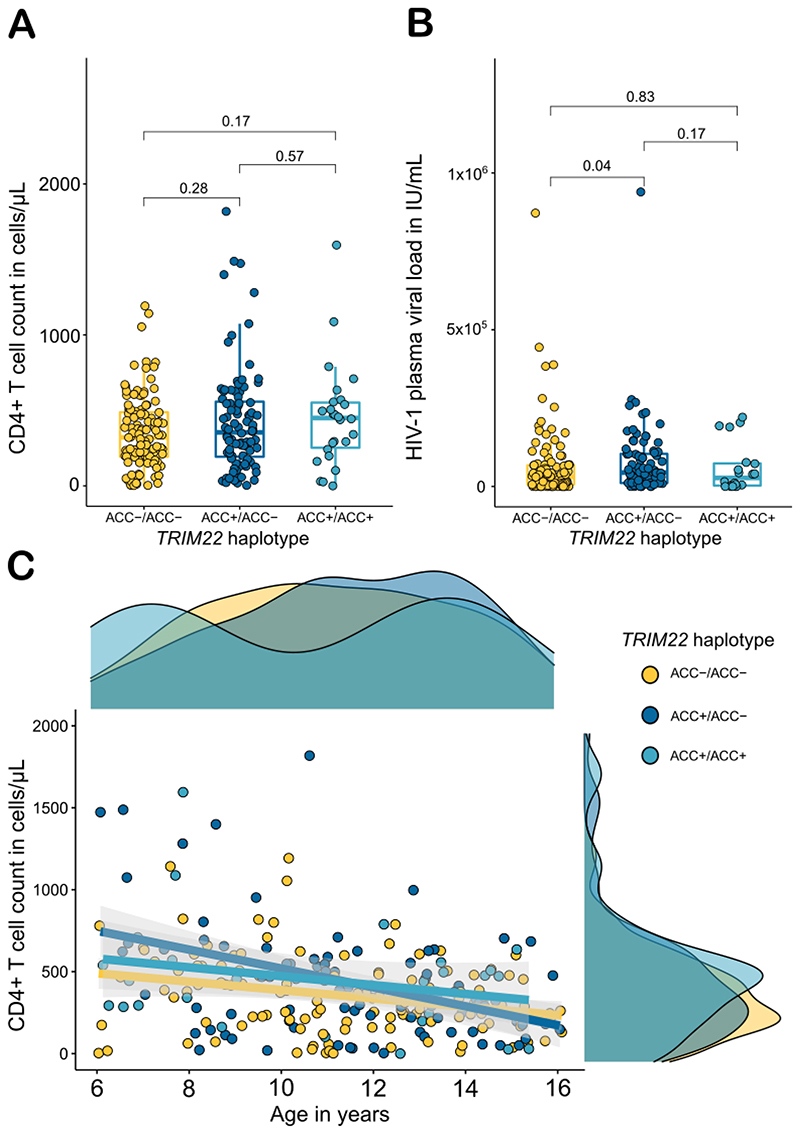
CD4+ T cell count and HIV viral load by homozygosity of *TRIM22* ACC haplotype. **Panels A and B** show box plots of CD4+ T cell counts and HIV-1 viral loads (y axes) by *TRIM22* haplotype homozygosity (x axes) with the interquartile range and median summarised. ACC+/ACC+ indicates that a participant is homozygous for this haplotype, ACC+/ACC- are heterozygous and ACC-/ACC- participants do not have this haplotype. *P* values are shown for pairwise MW tests for CD4+ T cell counts and HIV pVL. **Panel C** shows a scatter plot with linear regression lines and confidence intervals for ACC homozygosity with marginal density plots visualising the variables distributions.

We next determined the proportion of patients who were homozygous for *TRIM22* haplotype which included the reference allele at non-synonymous SNPs rs7935564(A), rs1063303(C) and rs61735273(C). After haplotype assignment there were 26 patients who were ACC homozygotes, i.e., these participants were homozygotes for the reference alleles at all three SNPs, having inherited ACC haplotypes from both parents. Ninety-two participants were heterozygous for this haplotype (inherited one ACC haplotype) and 123 did not have the ACC haplotype. ACC heterozygotes had a higher pVL than those who did not have ACC haplotypes (MW *P* = 0.04). ACC haplotype homozygotes had similar pVL and CD4 counts when compared to those without the haplotype (Fig. 1). *TRIM22* haplotypes were not associated with indicators of advanced HIV disease (table S3).

## Discussion

*TRIM22* genotype was not significantly associated with markers of disease progression or indicators of advanced disease in this study. Homozygosity for a haplotype of rs7935564(A) and rs1063303(C) did not significantly affect CD4+ T cell counts, HIV-1 pVL, or clinical variables - which contrasts with results reported for adults [16]. The rs1063303(G) allele is associated with enhanced expression of TRIM22 in vitro [26]. *TRIM22* RNA expression and HIV-1 plasma viral loads are negatively correlated in adults in the first year of infection, however, in chronic infection it is unknown if this association persists [14]. HIV-1 infection leads to differential expression of thousands of genes, and it is possible that there are significant differences in transcriptional profiles in children and adults with HIV prior to treatment – though this specific comparison remains unexplored [27].

Plasma viral loads are significantly higher in CWH who progress to advanced disease before two years of age (median of 1.1 million copies/mL), than in those who do not (median of 30 000 copies/mL) [5]. The ZENITH cohort enrolled individuals aged six years or older at HIV diagnosis and is likely to consist of slower disease progressors. Slower HIV disease progression in CWH is associated with immune phenotypes characterised by high CD4+ T cell counts, with low levels of immune activation despite high viral loads [8]. This phenotype is sustained by an expanded population of central memory T regulatory cells. Thus, viral loads may have stronger effects on disease progression in adults than in older CWH; and TRIM22, whose primary mechanism is limiting viral transcription, may have a more important role in the pathophysiology of HIV disease progression in adults than CWH [28]. Age did not vary significantly by *TRIM22* genotype or haplotype. Therefore, it seems unlikely that *TRIM22* genotype had a strong effect on early childhood survival in CWH.

The most consistent genetic associations with HIV-1 set-point pVL in adults are found in chromosome 3 and 6 [29]. In a genome-wide association study of HIV-1 infected adults from Botswana, SNPs in chromosome 11 (location of *TRIM22)* were not significantly associated with markers of disease progression [30]. This could mean that the previously reported associations of *TRIM22* with HIV-1 disease progression may not occur in African CWH [16,31]. HIV-1 disease progression represents a complex interaction between viral and host factors. It is therefore plausible that in this complex system the effects of *TRIM22* are small, and a much larger sample size may be needed to detect these effects [32].

## Supplementary Material

Supplementary Material

## Figures and Tables

**Table 1:** Summary results for demographics, disease progression markers and *TRIM22* genotypes in the ZENITH cohort

Total n (%)	241 (100)
Age in years [**median (IQR)**]	11.4 (9.1 - 13.5)
Male	121 (50.2)
Female	120 (49.8)
Stunting	38 (15.8)
Chronic diarrhoea	11 (4.6)
CD4+ count in cells/μL [**median (IQR)**]	342 (195 - 533)
CD4+ count < 200 cells/μL	66 (27.4)
HIV-1 viral load in lU/mL [**median (IQR)**]	34 199 (8211 - 90 662)
HIV-1 viral load < 1000 lU/mL	31 (14.9)
HIV-1 viral load 1000 - 99 999 lU/mL	132 (63.5)
HIV-1 viral load > 99 999 lU/mL	45 (21.6)
**rs200924168** - GG	239 (99.2)
**rs200924168** - GC	2 (0.8)
**rs200924168** - CC	0
**rs7935564** - GG	73 (30.3)
**rs7935564** - GA	108 (44.8)
**rs7935564** - AA	60 (24.9)
rs78484876 - TT	208 (86.3)
rs78484876 - TC	22 (9.1)
rs78484876 - CC	11 (4.6)
rs2291842 - TT	147 (61.0)
rs2291842 - TC	59 (24.5)
rs2291842 - CC	35 (14.5)
**rs1063303** - GG	96 (39.8)
**rs1063303** - GC	80 (33.2)
**rs1063303** - CC	65 (27.0)
**rs61735273** - CC	182 (75.5)
**rs61735273** - CT	41 (17.0)
**rs61735273**- TT	18 (7.5)

1 rs number refers to Reference SNP Cluster ID and functions as an accession number on the dbSNP database which is administered by the NCBI. Non-synonymous SNPs are highlighted in bold. Amino acid substitutions for non-synonymous SNPs in exon three: **rs200924168** = R150T and **rs7935564** = D155N; and exon four **rs1063303** = R238T and **rs617357273** = S244L. n = number of participants. IQR = interquartile range.
