# Supplementary material for: TRIM22 genotype is not associated with markers of disease progression in children with HIV-1 infection
Source: AIDS. Author manuscript; Available in PMC 2023 Aug 16. (PMC7614957; doi:10.1097/QAD.0000000000003053)
Supplement: Supplementary Material [file EMS182613-supplement-Supplementary_Material.docx]

**Supplementary materials**

**1. HIV viral load quantification**

Dan Hamieri-Bowen, Louis Marie-Yindom, and staff at the Biomedical Research and Training Institute measured HIV-1 viral load. Viral load quantification was done using RealStar Qualitative HIV RT-PCR Kit 1.0 (Altona Diagnostics, Hamburg, Germany) and quantified with the QuantStudio 3 Real-Time PCR system (Applied Biosystems, Waltham, MA). Viral load was converted to international units (IU) using the following formula.

$\boldsymbol{Viral load(sample)[IU/mL]=(Volume(Eluate)[\mu m] \times Viral load(Eluate)[IU/\mu m])\div(Sample Input[mL]}$**)**

**2. DNA extraction protocol**

DNA extraction was done by laboratory personnel at the Biomedical Research and Training Institute, Harare, Zimbabwe.

**Day 1**

**Red blood cell lysis**: 10mL of whole blood was added to a 50mL falcon tube. The sample was vortexed and then 30mL of Tris 20Mm- Ethylenediaminetetraacetic acid (EDTA) 5mM (TE 20-5) was added up to volume of 40mL, the tube was then inverted several times and incubated on ice for 50min. Following this the sample was centrifuged at 3500rpm for 15min and the supernatant then removed. The remaining pellet was then vortexed. TE was then added to a total volume of 40mL and the sample then vortexed and centrifuged at 3500rpm for 15min. This step was repeated until the remaining pellet was white.

**White blood cell lysis:** After the red cells were adequately lysed the remaining white pellet was vortexed until no cell clumps remained. 2mL of TE 20-5 was added followed by 100µL of 20% solution of sodium dodecyl sulphate. To digest remaining proteins 20µL of proteinase-K (10mg/mL) was added and the sample placed in a shaking water bath for overnight incubation at 42ºC.

**Day 2**

**Ethanol precipitation:** After the overnight incubation the sample was removed from the water bath and cooled to room temperature. 2.5mL of 7.5M ammonium acetate was added and the sample placed on ice. Next, 10mL of chilled 100% ethanol was added followed by centrifugation at 5000rpm for 30min. The supernatant was then removed, and 4mL of sterile Tris 20mM-EDTA 5mM-NaCl 0.2M (TE 20-5 NaCl 0.2M) was added, followed by vortexing the sample. The sample was then incubated at 42ºC until the DNA was fully dissolved into the solution. The DNA was then re-precipitated in 8mL of 100% ethanol, followed by centrifugation at 5000rpm for 30min. The supernatant was then removed and allowed to stand until no fluid remained.

The DNA sample was then resuspended in 150µL of Tris 20mM-EDTA 1 Mm (TE 20-1) and incubated at 58ºC to dissolve the sample completely. Samples were then stored in 1.5mL Eppendorf’s at 4ºC. DNA concentration and quality were quantified by spectrophotometry (Nanodrop 2000, Thermo Fisher Scientific, Waltham, USA)

**3. PCR and sequencing of *TRIM22***

Amplification primers were designed to flank exons 2 - 4 using the *TRIM22* reference sequences NC_000011 from the NCBI database. Non-specific amplification producing multiple products was a significant problem during optimisation of these protocols. This can be attributed to the presence of multiple paralogues of *TRIM22* in the human genome. To rectify this, touchdown amplification protocols were developed for polymerase chain reaction (PCR). Single step, touchdown PCR reactions were optimised, and separate inner sequencing primers were designed to sequence individual exons within the larger amplicons. All primers were synthesised by Life Technologies (Thermo Fisher Scientific, Waltham, MA)

Table S1: Primers for amplifying and sequencing *TRIM22*

| **Primer name** | **5’ – 3’ oligo sequence** | **Binding site** ^1^ |
| --- | --- | --- |
| **Primers for PCR** | | |
| T22 E2_4F_2 | GGGTTACACGAAGCTCTTGG | 6292 - 6311 |
| T22 E2_4R_2 | TGAATGGGAAGGGAAATTCC | 9137 - 9157 |
| **Primers for sequencing** | | |
| T22E2-3F SEQ PRI | ACTGCAGGAGTTTGTGACCAAG | 6582 - 6603 |
| T22E2-3R SEQ PRI | CCTTCCTCTTTGCTGTGCTAG | 7833 - 7853 |
| T22E4F SEQ PRI | CAGGCTCATACAAAGCAG | 8663 - 8680 |
| T22E4R SEQ PRI | GACATGAAATGGTTCCTTTGGCC | 9059 - 9081 |

^1^ Binding site in reference sequence NC_000011. Reverse primers were ordered as reverse complement sequences of those listed in the table.

**Master mix and cycling conditions for PCR**

All PCRs were done using the Advantage 2 PCR kit (Takara, Kusatsu, Japan), all PCR, PCR purification and pre-sequencing reactions were performed on an Alpha Cycler 4 (PCRmax Limited, Staffordshire, United Kingdom).

## Master Mix per reaction:

16.5µL PCR grade H_2_0 (sterile, RNA and DNA free)

2.5µL of Advantage 10X Buffer SA

2.0µL of forward primer at 10ng/µL

2.0µL of reverse primer at 10ng/µL

2.0µL of 2.5Mm mixed deoxyribonucleotide triphosphate (dNTP) (Takara)

0.5 µL of 50X Advantage 2 polymerase mix (Takara)

Then add 1.0µL of 50ng/µL gDNA sample for a total of 25µL per reaction

Thermocycler settings for touchdown PCR of *TRIM5* and *TRIM22*

| **PCR step** | ***T22* exon2-4 ^2^** |
| --- | --- |
| 1. Denaturation | 95 ^o^C – 60s |
| 2.1 Denaturation | 95 ^o^C – 30s |
| 2.2 Annealing | 67 – 57 ^o^C – 30s |
| 2.3 Elongation | 68 ^o^C – 3min30s |
| 3.1 Denaturation | 95 ^o^C – 30s |
| 3.2 Annealing | 57 ^o^C – 30s |
| 3.3 Elongation | 68 ^o^C – 3min30s |
| 4. Final elongation | 68 ^o^C – 5min |

^1^ For each stage of the PCR temperature is shown in degree Celsius with the time for each step reported in minutes and seconds.^2^ *T22* = *TRIM22*.

Steps 2.1 to 2.3 were repeated for 10 cycles, decreasing the annealing temperature by 1 ^o^C per cycle. Following this, steps 3.1 to 3.3 were repeated for 25 cycles with the same annealing temperature for a total of 35 cycles of amplification.

Post-PCR product visualisation and purification

After PCR, products were separated via gel electrophoresis on a 1% agarose, Tris-acetate-EDTA (TEA) gel stained with ethidium bromide for 25min at 110V. Products were then visualised under ultraviolet light in a Gel-doc against a 1kb hyperladder (Bioline). PCR products were then purified for sequencing using an

**Exo-Sap master mix per reaction**:

1. Recombinant exonuclease I (New England Biolabs, Ipswich, USA) - 0.1µL per reaction

2. Recombinant shrimp alkaline phosphatase (New England Biolabs) - 0.5µL

3. PCR grade H_2_0 - 0.4µL

1.0µL of the EXO-SAP mix was then added to each PCR product and incubated on a thermocycler as follows:

1 cycle 37 ^o^C for 45min

1 cycle 80 ^o^C for 25min

Sanger pre-sequencing reaction

After post-PCR processing PCR products were pre-sequenced via Sanger sequencing using the Big Dye Terminator (BDT) v3.1 Cycle sequencing kit (Thermofisher, Waltham, MA). Template negative controls were carried through from PCR through product purification and sequencing to ensure that contamination between wells did not occur in a sequencing plate.

**The BDT master mix per reaction**:

1. BDT 5X buffer - 2.08µL

2. BDT v1.1 V3.1 - 0.25µL

3. PCR grade H_2_0 - 5.36µL

4. Primer (10 µM) - 0.32µL

5. PCR product - 2.0µL

**Pre-Sequencing reactions were performed on a thermocycler as follows**:

1. 96^o^C – 1min

2. 96^o^C – 10s

3. 50^o^C – 5s

4. 60^o^C – 4min

Post-sequencing product purification

Excess oligonucleotides were removed from pre-sequenced PCR products using ethanol precipitation as follows:

5µL of 125mM EDTA was added to each sample well containing 2µL of sequenced PCR products and incubated at room temperature for 5min. 40µL of 100% ethanol was added to each well and incubated at -20^o^C for 1 hour. Following incubation, DNA salts were centrifuged at 3000g for 30min at 4^o^C on a Heraeus 40 centrifuge (Thermofisher). After centrifugation, uncovered sequencing plates containing the precipitated DNA salts were inverted and briefly centrifuged at 100g. 40µL of 70% ethanol was added into each well and centrifuged at 3000g for 15min at 4^o^C. The uncovered sequencing plates were again inverted and centrifuged at 200g for 1min. The precipitated DNA salts were resuspended in 10µL of Hi-Di Formamide. Samples were sequenced the same day or stored at -20^o^C. Sequencing was performed on a 3730XL ABI sequence analyser at the Medical Research Council Weatherall Institute for Molecular Medicine (University of Oxford, United Kingdom).

Sequence electropherograms for *TRIM22* were analysed in Geneious 2019.2 (Biomatters, Auckland, New Zealand) [1]. Forward and reverse reads for each exon were collected, trimmed for quality, and assembled into a contiguous sequences per participant. The participants’ assembled contigs were aligned to the NC_000011 reference sequence. Single nucleotide polymorphisms were identified at a 35% heterozygosity threshold according to Applied Biosystems recommendations and identified in relation to the dbSNP database administered by the NCBI [2]. Haplotype inference was done using Arlequin version 3.0 [3]

**4. Comparison of summary statistics for ZENITH cohort versus genotyped sample**

Summary statistics for the genotyped sample of participants was similar to the larger cohort.

Table S2: Summary statistics for ZENITH cohort and the genotyped sample [4]

|  | ZENITH cohort | Genotyped sample |
| --- | --- | --- |
| Number | 385 | 241 |
| Median Age (years) | 11.0 | 11.4 |
| Sex - male % | 48 | 50.2 |
| Sex - female % | 52 | 49.8 |
| CD4+ T cell count (Median) | 375 | 342 |
| CD4+ T cell count (IQR)^1^ | 215 - 599 | 193 – 533 |
| HIV-1 viral load (Median) | 36 047 | 34 199 |
| HIV-1 viral load (IQR)^1^ | 13 377 - 86 970 | 8211 – 90 662 |

^1^IQR = interquartile range

**5. Results of logistic regression models**

Results from logistic regression modelling of indicators of advanced disease are summarised in table S3. Age was significantly associated with CD4+ T cell counts and therefore included as an additional variable in multivariate models of *TRIM22* genotype/haplotype associations. Multivariate models were not analysed for associations of stunting and chronic diarrhoea.

Table S3: Association of *TRIM22* genotype with indicators of advanced disease

1) aOR = adjusted Odds Ratio. 2) HIV-1 pVL analysed in log10(HIV viral load in IU/mL). Cells highlighted indicate where *P* values met statistical significance. Blank cells indicate where models did not converge (results include infinity values) or where multivariate modelling was not done.

**6. References**

1 Kearse M, Moir R, Wilson A, Stones-Havas S, Cheung M, Sturrock S, *et al.* **Geneious Basic: an integrated and extendable desktop software platform for the organization and analysis of sequence data**. *Bioinforma Oxf Engl* 2012; **28**:1647–1649.

2 Applied Biosystems. **Detection and Quantification of Sequence Variants from Sanger Sequencing Traces**. *Life Technol Corp* 2013; :1–13.

3 Excoffier L, Laval G, Schneider S. **Arlequin (version 3.0): An integrated software package for population genetics data analysis**. *Evol Bioinforma Online* 2007; **1**:47–50.

4 McHugh G, Simms V, Dauya E, Bandason T, Chonzi P, Metaxa D, *et al.* **Clinical outcomes in children and adolescents initiating antiretroviral therapy in decentralized healthcare settings in Zimbabwe**. *J Int AIDS Soc* 2017; **20**:1–9.
